# Identification of Drug Resistant Mutations in HIV-1 CRF07_BC Variants Selected by Nevirapine *In Vitro*

**DOI:** 10.1371/journal.pone.0044333

**Published:** 2012-09-12

**Authors:** Hao Wu, Hao-Jie Zhang, Xiao-min Zhang, Hui-fang Xu, Ming Wang, Jian-dong Huang, Bo-Jian Zheng

**Affiliations:** 1 Department of Microbiology, University of Hong Kong, Hong Kong SAR, Guangzhou CDC, Guangzhou, China; 2 Department of Biochemistry, University of Hong Kong, Hong Kong SAR, Guangzhou CDC, Guangzhou, China; 3 University of Hong Kong, Hong Kong SAR, Guangzhou CDC, Guangzhou, China; University of Pittsburgh, United States of America

## Abstract

Since the antiretroviral therapy (ART) was introduced to patients infected by human immunodeficiency virus (HIV), the HIV related mortality and morbidity have been significantly reduced. The major obstacle for long-term successful anti-HIV treatment is the emergence of drug resistant mutants. Current data of drug resistance was mainly obtained on HIV-1 subtype B but rarely on non-B virus, even more rare with newly emerged circulating recombinant forms (CRFs). The lack of such data limits the rational management of ART for the increasing number of patients infected by non-subtype B virus. In this study, a HIV-1 CRF07_BC strain CNGZD was isolated from a HIV patient and its genome was sequenced and deposited in GenBank (JQ423923). Potential drug resistant mutants of this CRF07_BC virus strain were selected in PBMCs cultures in the presence of Nevirapine (NVP), which is the most frequently used antiretroviral drug in China. Four combination profiles of mutations were identified in the NVP-selected mutants, which were initiated with A98G, V108I, Y181C and I135T/I382L and followed by more than two other mutations at the end of the selections, respectively. A total of seven previously reported mutations (A98G, V106M, V108I, I135T, Y181C, V189I, K238N) and seven novel mutations (P4H, T48I, I178M, V314A, I382L/V, T386A) in the reverse transcriptase gene were found in these NVP-selected mutants. Phenotypic analysis in the NVP-selected mutants showed that all the mutations, except P4H, contribute to NVP resistance. Among them, V106M and Y181C reduce NVP susceptibility for more than 20-fold, while the other mutations cause less than 20 folds drug resistance. Although the information obtained in this *in vitro* selection study may not fully cover resistant mutations which will actually occur in patients, it has still provided useful information for rational management of ART in patients infected with HIV CRF_BC subtype.

## Introduction

Circulating recombinant forms (CRFs) are the virus with recombinant genomes from different subtypes, which have been detected as epidemic strains and displayed as their identifying numbers and with letters presenting the involved subtypes [1]. Studies of HIV-1 subtypes and recombinants distributions in worldwide have showed that the proportion of CRFs increased from 12% in 2000–2003 to 16% in 2004–2007 and CRFs may account for more and more infection cases in the coming years [2]. In China, CRF07_BC was originally found in Yunnan province and spread out through one of the heroin trafficking route from Yunnan to the northwestern provinces Sichuan, Gansu, Ningxia and Xinjiang, and then across the border to Kasakhstan [3], [4]. It has been reported that CRF07_BC became the most prevalent subtype in China (50.20%) in 2004 [5] and the most dominant subtype in the newly diagnosed patients in Beijing (32.5%) in 2006–2007 [6].

Nevirapine (NVP), the first generation of nonnucleoside reverse transcriptase (RT) inhibitor (NNRTI), is widely used with nucleoside RT inhibitor (NRTI) as the first line antiretroviral regimens, while NVP-containing highly active antiretroviral therapy (HAART) is the most popular drug combination in China [7]. Similar to the other NNRTIs, NVP binds to RT through a hydrophobic pocket adjacent the active site of the enzyme. NVP shows low genetic barrier for resistant mutations and resistant mutation profiles of NVP always overlap with those of other NNRTIs [8]. Furthermore, genetic diversity in different subtypes or CRFs may affect the drug resistance development in patients [9]. Thus far, the limited information for potential drug resistance of CRF07_BC was based on the investigation in treatment naïve patients but not from treatment failure patients [5]. The genotypes and phenotypes of the potential drug-resistant mutations in treatment naïve patients still remain unclear after receiving HAART. With the increasing cases of CRF07_BC infection, more and more patients will be given antiviral therapy. To improve managements of NVP-containing antiretroviral therapy for these patients, it is urgent to know the NVP resistant mutation profiles in CRF07_BC. However, in the current studies based on treatment failure patients, drug-resistance cannot be diagnosed before the treatment failure, which always needs several years.

In the present study, we cultured clinical isolated CRF07_BC virus with increasing concentration of NVP in PBMC to induce NVP associated mutations *in vitro* and further analyzed phenotypes of these mutations by comparing levels of NVP resistance among the induced CRF07_BC mutants. The study results will provide important information of NVP resistant mutations for improving the management of NVP containing antiviral treatment for patients carrying CRF07_BC virus.

## Materials and Methods

### Virus Strain and Compound

The virus strain of CRF07_BC was isolated from a 32-year-old female injection drug user (IDU) living in Guangzhou. Patient’s blood sample was collected by Guangzhou CDC in 2007 when the patient was asymptomatic and did not start antiviral treatment. Virus was isolated by co-culture of patient’s PBMC with healthy PBMC in RPMI 1640 supplemented with 10% fetal bovine serum and antibiotics and 1 µg/ml interleukin-2 (IL-2) and was named as CNGZD. Biological clones of CNGZD were also prepared using a limiting dilution protocol [10]. The isolated virus was quantified by blue focus units (BFU) assay as described previously [11], [12]. Briefly, virus stock was 3 to 10-fold serially diluted by DMEM complete culture medium containing 25 µg/ml DEAE and inoculated to TZM-bl cells seeded in a 96 well plate. The infected cells were incubated at 37°C for 48 hrs and stained with X-Gal solution. After two hours incubation at 37°C, the blue cells were easily visualized and counted under light microscopy. NVP was provided by Boehringer Ingelheim (Germany), which was dissolved in DMSO and stored at –20°C until use.

### Full Viral Genomic Sequence Analysis

Viral RNA was extracted from primary virus culture supernatant by QIAamp Viral RNA Mini Kit (Qiagen, Hilden, Germany). Virus cDNA was synthesized from virus RNA using SuperScript™ II Reverse Transcriptase (Invitrogen). Nearly 9.0 kb full length genome of CNGZD was recovered by four independent PCR reactions using 4 paired primers, in which the PCR products at size of about 2.0–2.5 kb were overlapping the whole virus genome except long terminal repeat (LTR) fragment. Each segment of viral PCR products was subcloned into pMD18-T Simple Vector (TaKaRa) using ligation kits (TaKaRa). Five plasmids for each PCR segment were prepared for cycle sequencing PCR. The sequence electrograms of each sample were aligned using Staden Package [13] and all sequences were assembled by Vector NTI advance 10 (Invitrogen). Virus coding sequences (CDSs) were pointed out by online software HIV Sequence Locator. Recombinant Identification Program (RIP) was used for analysis of HIV-1 subtypes and recombinations. REGA HIV-1 Subtyping Tool - Version 2.0 was used for confirmation of virus subtypes [14]. Phylogenetic analysis was performed to figure out the subtype of virus strain. Genotypic resistance interpretation was also done using Stanford University HIV Drug Resistance Database HIVdb program (version6.0.11) (http://hivdb.stanford.edu). The viral genome sequence was annotated and deposited into GenBank (accession number: JQ423923).

### *In Vitro* Selection of CRF07_BC Variants Resistant to NVP

Primarily isolated virus strain and two biological clones of CNGZD were used for *in vitro* selection of virus variants resistant to NVP in three and two independent cultures, respectively, according to the methods described previously [15]. Briefly, PBMCs (1×10^6^) were initially infected with 2×10^5^ BFU/well of the virus in a 24-well tissue culture plate (in quintuple) and cultured in 1 ml medium containing diluted NVP. The starting and ending NVP culture concentration was 5 nM and 64 µM, respectively. One NVP untreated well was included as negative control. At day 3, 7 and 10, virus titers in culture supernatant of each well were detected by BFU assay. Once the BFU number in NVP treated cultures was more than 20% of the untreated control, the drug concentration was increased to 2-fold. While the BFU number in NVP treated cultures was less than 5% of the untreated control, restart the selection. The whole selection was carried out for 25 passages. Passages were performed with supernatant of preceding passage and conducted in the same rules mentioned above. Culture supernatants of passage 5, 10, 15, 20 and 25 were saved for genotypic and phenotypic resistance analysis.

### Genetic Resistance Analysis in Resistant Variants

Viral RNA was extracted by QIAamp Viral RNA Mini kit (Qiagen, Hilden, Germany) and viral cDNA was reverse-transcribed using SuperScript™ II Reverse Transcriptase (Invitrogen) according to the manufacturer’s protocol. The entire protease gene and the first 440 codons of RT gene were amplified from cDNA by high fidelity AccuPrime™ Taq DNA Polymerase (Invitrogen) using primers AV150 and polM4 as described previously [16], [17]. PCR products were purified using QIAquick PCR Purification Kit (Qiagen) and analyzed on 1% TBE agarose gel to confirm their size and purity. The purified PCR products were direct sequenced in both directions using the Big Dye terminator v3.1 cycle sequencing kit (Applied Biosystems). The sequence results were aligned with Staden Package [13] and submitted to the Stanford University HIV Drug Resistance Database HIVdb program (version6.0.11)(http://hivdb.stanford.edu) for the interpretation of genotypic resistance as described previously [18].

### Phenotypic Analysis of NVP Resistant Variants

Phenotypic analysis was performed in TZM-bl cells containing luciferase reporter gene induced by HIV infection as described previously [19]. Briefly, the TZM-bl cells were infected with 400–600 BFUs/well of the virus and then added with 7 serial dilutions of NVP in triplicate. After 48 hrs culture, luciferase activities were tested by luciferase assay system (Promega) according to the manufacturer’s protocol. The 50% effective concentration (EC_50_) of NVP was determined according to the curve which was plotted by the percentage of reduction of luciferase activity versus serial NVP concentrations. NVP resistance of virus variant was expressed as the fold change (FC) of NVP EC_50_. The NVP susceptibility fold-change of virus variants was defined as the ratio between the EC_50_ of drug resistant variants with certain mutations and the EC_50_ of virus without the certain mutations.

## Results

### Analysis of the Viral Genome Sequence of CNGZD Isolated from the Patient

**Figure 1:**
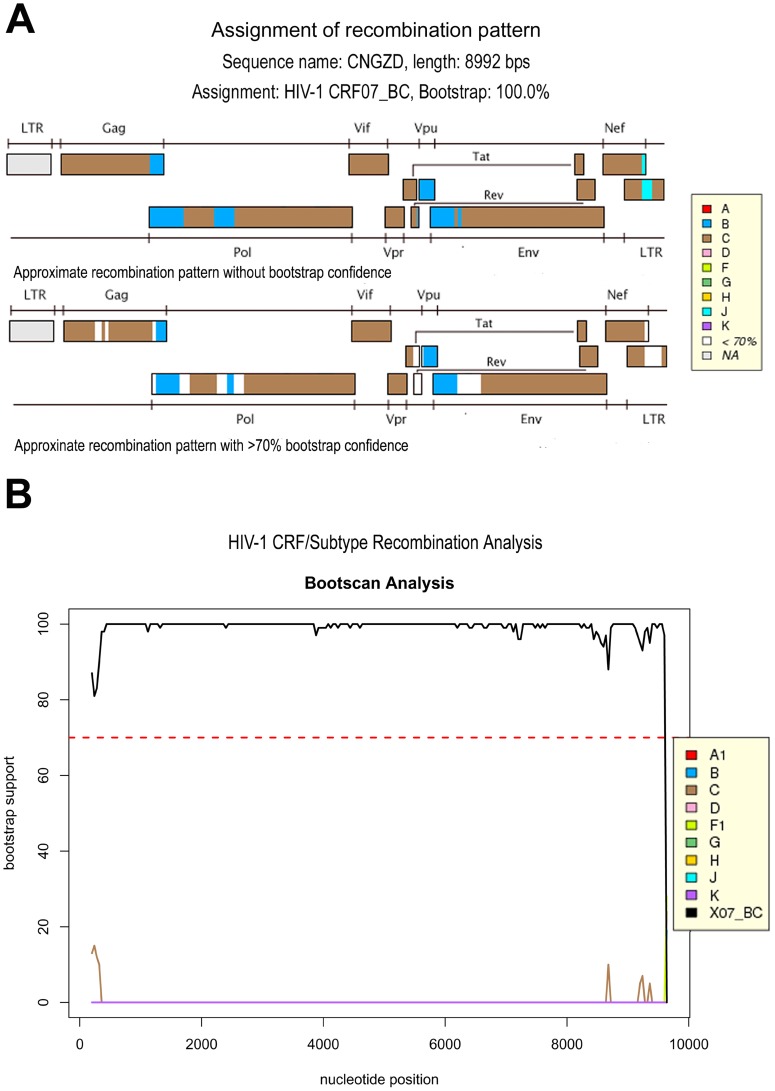
Subtyping analysis of the genome of CNGZD using Rega HIV-1 subtyping tool (version 2.0). The recombination pattern was determined by comparing CNGZD sequence with reference sequences of HIV-1 pure subtypes A, B, C, D, F, G, H, J, K. Five DNA segments from subtype B were found to be integrated into the genomic backbone of subtype C by phylogenetic methods of the Rega HIV-1 subtyping tool, which can identify the subtype of a specific sequence. The result was displayed as a virus genomic structure figure with subtype components (A). For subtype recombination analysis, reference sequences of different pure subtypes and CRFs were further applied for the comparison of the virtually full-length sequence of CNGZD by bootscanning methods. Bootscan analysis showed that CNGZD belonged to CRF07_BC (B).

**Figure 2:**
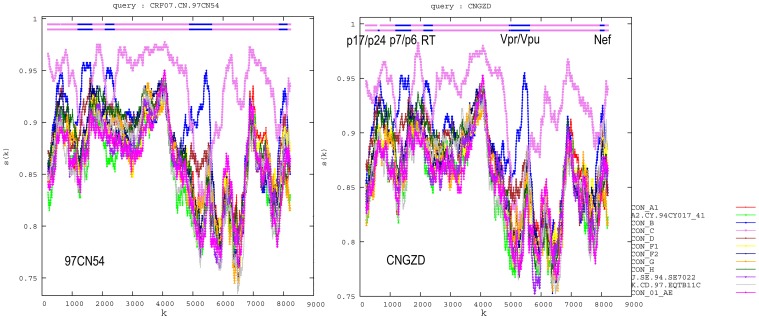
Analysis of nearly full length genome of CNGZD using Recombinant Identification Program (RIP) (version 3.0). Mosaic structures of subtype B (in blue) and C (in violet) in both CNGZD and the reference strain of CRF07_BC (97CN54) were figured out by software RIP and the recombinant profile of CNGZD was similar to the reference strain 97CN54. Five segments from subtype B were found in subtype C backbone and the recombination points located at positions in p17/p24, p7/p6, RT, Vpr/Vpu and nef regions of CNGZD and 97CN54, respectively. Window size: 200; threshold for statistical significance: 90%.

**Figure 3:**
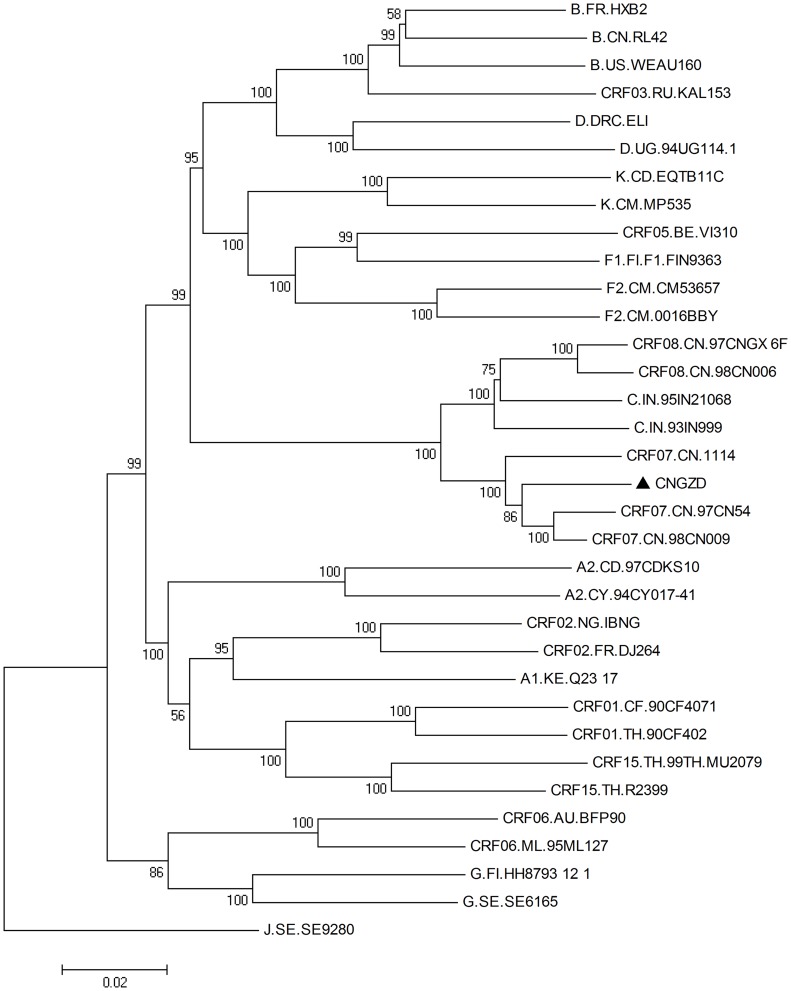
Phylogenetic analysis of clinical isolated virus strain CNGZD. A neighbor joining phylogenetic tree was built by Mega 4.1 based on full length sequences of clinical isolated virus strain CNGZD and different pure subtypes and CRFs. The Kimura 2-parameter substitution model was used and bootstrap values (1000 replicates) higher than 50 were shown next to the nodes of the tree. J.SE. SE9280 with long distance to CRF07_BC was selected as an outgroup in the phylogentic tree. Subtype references were downloaded from the Los Alamos HIV database (http://hiv-web.lanl.gov). CNGZD located in a clade with the other CRF07_BC isolates. Bar: 0.002 substitutions per site.

The 9.0-kb full length viral genome of HIV strain CNGZD was amplified to obtain the viral genome except the LTR at the 5′ terminal. The sequence contained all the known viral genes, including *gag, pol, vif, vpr, tat, rev, vpu, env* and *nef* coding for viral proteins p17, p24, p2, p1, p6, PR, RT, IN, Vif, Vpr, Tat, Rev, Vpu, gp120, gp41 and Nef, respectively (Table 1). The results of subtyping analysis showed that CNGZD belonged to HIV-1 CRF07_BC as determined in both phylogenetic and bootscanning methods using Rega HIV-1 subtyping tool (version 2.0) (Figure 1). Mosaic structures from different subtypes in CNGZD were confirmed by RIP and compared with that of CRF07_BC reference strain 97CN54 (Figure 2). The sequence of CNGZD was aligned with near-full-length consensus sequences of subtypes A1, A2, B, C, D, F1, F2, G, H, CRF01_AE in HIV database. The distance value between the sequence of CNGZD and the subtype marked by different color was calculated at each window size. The subtype with the highest similarity to CRF07_BC in every moving window was presented on the top of the graph. For CNGZD and CRF07_BC reference strain 97CN54, five segments from subtype B were found in subtype C backbone and the recombination points located in similar positions at p17/p24, p7/p6, RT, Vpr/Vpu, nef regions (Figure 2). The phylogenetic tree also showed that CNGZD belonged to CRF07_BC clade together with other clinical isolated virus (Figure 3). Pre-existing drug-resistant mutations in PR and RT genes of CNGZD were analyzed by Genotypic Resistance Interpretation Algorithm. No major or minor PR mutations and no NRTI mutations were found in CNGZD, but a NNRTI mutation V179E was detected, which was predicted to cause low-level resistance to NVP, efavirenz (EFV) and delavirdine (DLV) according to the comment of Stanford University HIV drug resistance database [20]. Thus, CNGZD was considered as a representative member of CRF07_BC and used for the *in vitro* selection to investigate the NVP mutation profile in clinical isolated CRF07_BC virus.

**Table 1:** Genomic regions covered by sequences of CNGZD.

CDS	Positionin HXB2	Amino acidin HXB2	Position in CNGZD
NCR	654–789	7–52	1–136
Gag	790–2292	1–501	137–1624
p17	790–1185	1–132	137–523
p24	1186–1878	1–231	524–1216
p2	1879–1920	1–14	1217–1252
p7	1921–2085	1–55	1253–1417
p1	2086–2133	1–16	1418–1465
p6	2134–2292	1–53	1466–1624
Pol	2085–5096	1–1004	1417–4428
p2p7p1p6	1879–2292	1–138	1217–1624
GagPolTF	2085–2252	1–56	1417–1584
Protease	2253–2549	1–99	1585–1881
RT	2550–3869	1–440	1882–3201
RNase	3870–4229	1–120	3202–3561
Integrase	4230–5096	1–289	3562–4428
Vif	5041–5619	1–193	4373–4954
Vpr	5559–5850	1–98	4894–5184
Tat1	5831–6045	1–72	5165–5379
Rev1	5970–6045	1–26	5304–5379
Vpu	6062–6310	1–83	5396–5641
gp160	6225–8795	1–857	5559–8132
gp120	6225–7757	1–511	5559–7073
gp41	7758–8795	1–346	7074–8132
Tat2	8379–8469	1–31	7695–7785
Rev2	8379–8653	1–92	7695–7990
Nef	8797–9417	1–207	8133–8750
LTR3	9086–9673	1–184	8419–8992

All the coding sequences (CDSs) in CNGZD were pointed out and the corresponding position of each CDS in the virus genome was indicated in both submitted sequence and HIV-1 reference strain HXB2 by the online software HIV Sequence Locator.

### NVP-associated Mutations and Combination Profiles of CRF07_BC Selected *in vitro*

**Figure 4:**
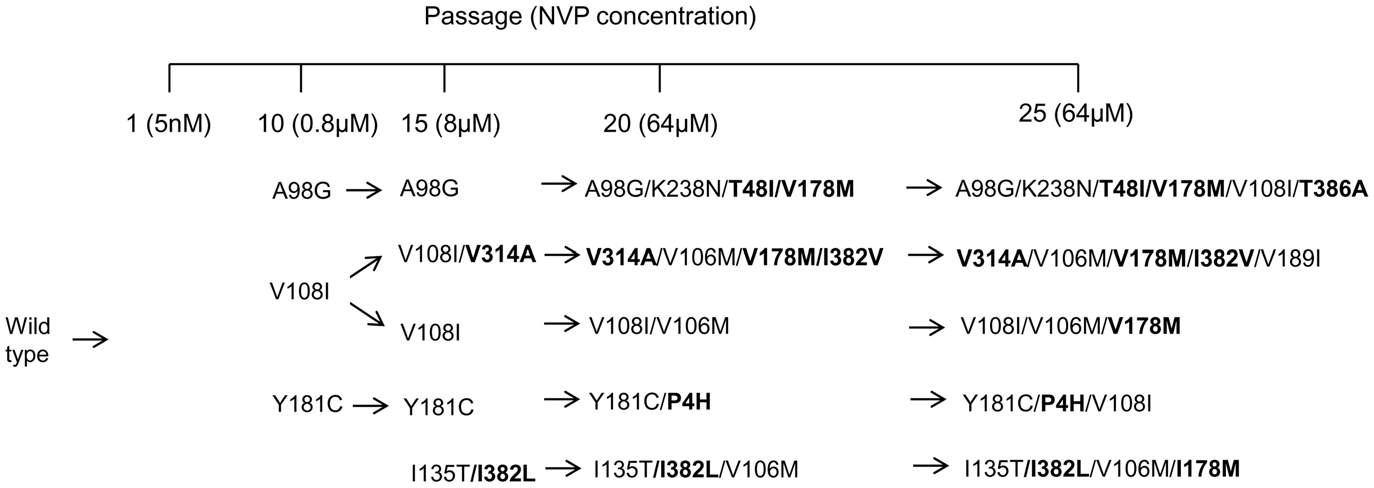
Combination profiles of mutations selected by NVP in CRF07_BC. Mutations that were bolded are novel mutations. Four different combination profiles initialed with mutations A98G, V108I, Y181C and I135T with I382L were selected by NVP in CRF07_BC.

CRF07_BC strain CNGZD was passaged with increasing concentration of NVP to select the virus variants resistant to the drug. No any mutation was detected in passage 5 of each parallel culture. After 25 passages of PBMC cultures in the presence of gradually increasing concentration of NVP, four combination profiles of mutations, which were initialed by three previously reported mutations A98G, V108I, Y181C [21], [22], and reported mutations I135T [23] together with a novel mutation I382L, were identified (Figure 4). In these combination profiles, three previously reported mutations K238N, V106M, V189I [24], and 6 novel mutations T48I, I178M, T386A, V314A, I382V and P4H appeared gradually and formed combinations of up to 3–5 mutations at the end of the selection. Among them, I178M appeared in four parallel cultures, while V106M was selected in three independent virus cultures together with I178M. V108I was also found in three selected mutants as initial or non-initial mutation in our study. As summarized in Table 2, a total of seven known mutations and seven novel mutations were identified in NVP-selected variants. According to the comments given by Stanford University HIV-1 Resistance Database, mutations V106M and Y181C can cause high level resistance to NVP, while mutations A98G and V108I just result in low-level reduction in susceptibility to NVP. Another mutation K238N alone is not a common mutation selected by NNRTI treatment and its effect on resistance to NVP is still unknown [25]. Seven novel mutations P4H, T48I, I178M, V314A, I382L/V and T386A have not been reported to cause NVP resistance. All these mutations, including the pre-existing congenital NNRTI minor mutation V179E, persisted to the end of this *in vitro* study. These results showed that NVP-selected mutations and their combination profile in HIV-1 subtype CRF07_BC were complicate and diverse.

**Table 2:** Mutations developed in RT gene of CRF07_BC strain CNGZD during the *in vitro* selection with increasing concentrations of NVP.

**NVP associated mutations predicted by HIVdb program**									
	98	106	108	135	181	189	238		
B, C, CRF07_BC cons	A	V	V	I	Y	V	K		
NVP	G	M	I	T	C	I	N		
**Novel mutations**									
	4	48	178	314	382	386			
B, C, CRF07_BC cons	P	S,T	I	V	I	T			
NVP	H	I	M	A	L/V	A			

The conserved amino acid at position 48 of subtype B is S while that in subtype C and CNGZD is T.

### Phenotypic Analysis of CRF07_BC Virus Variants Selected by NVP

**Figure 5:**
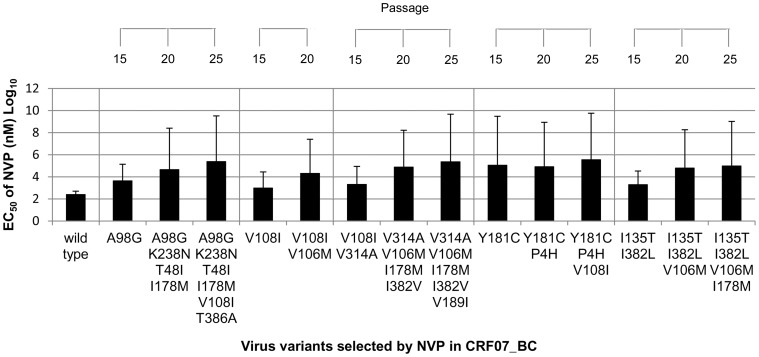
Phenotypic analysis of CRF07_BC virus variants selected by NVP. The NVP EC_50_ of CRF07_BC virus variants with different mutations were shown. TZM-bl cells were infected with virus variants and treaded with serial diluted NVP. After 48 hrs incubation, luciferase activities representing HIV infection were tested. The EC_50_ NVP was estimated from the standard curve plotted by the percentage of reduction of luciferase activity and serial concentrations of NVP.

To analyze the phenotypes of NVP-selected CRF07_BC virus variants, the NVP susceptibility of 14 virus variants selected in the *in vitro* culture was tested in TZM-bl cell cultures (Figure 5). Among 4 variants with the initial mutations A98G, V108I, Y181C, and I135T/I382L respectively, the variant with the mutation Y181C showed the highest resistance to NVP, which resulted in about 450 folds increase of NVP resistance. The EC_50_ of NVP in the variants with the mutations A98G or two other mutations I135T/I382L increased for about 17 or 7 folds, while that in the variant with mutation V108I increased for less than 4 folds. In one of the parallel culture, the initial mutation V108I disappeared later in the subsequent selection cultures with higher concentration of NVP. In the variants appeared subsequently in the presence of increasing concentrations of NVP, the variant with additional mutation V106M caused about 22–32 folds increase of NVP resistance, while the variants with additional mutations K238N/T48I/I178M and V108I/T386A showed about 5–10 folds increase of NVP resistance. The NVP resistance of the variants with the other additional mutations V314A, V189I, I178M and I178M/I382V increased for less than 4 folds, but the mutation P4H was not related to NVP resistance. The drug resistant effect of single mutation was further analyzed by comparing NVP resistance in variants with different combinations of mutations. For example, initial or subsequent mutation V108I was associated with 3.5 or 3.1 folds increase of NVP resistance, while mutations V108I/T386A accounted for 5 folds increase of NVP resistance. Thus, NVP resistance of the variant with mutation T386A alone should increase less than 4 folds as compared to wild type. Similarly, the variants with additional K238N/T48I/I178M, I178M/I382V and I135T/I382L resulted in about 10, 3.5 and 7.3 folds increase of NVP resistance, respectively. Therefore, the novel mutations I178M and I382L, and the reported mutation I135T would be associated with low level of NVP resistance (<4 folds increase).

Based on the results of NVP susceptibility, NVP-selected mutations were classified to high (over 20-fold), medium (between 4 to 20-fold) and low (less than 4-fold) level resistance to NVP. As summarized in Table 3, two reported mutations V106M and Y181C were characterized to be associated with high level of NVP resistance, while the other 5 known mutations A98G, K238N V108I, I135T and V189I were classified as medium or low level of NVP resistance. Among seven novel mutations, one (P4H) did not show any NVP resistant effect and the other 6 were associated with low level of NVP resistance. Even in the combination of two mutations, I135T/I382I was associated with median level of NVP resistance but I178M/I382V only gave rise to low level of NVP resistance.

**Table 3:** Characterization of NVP resistance mutations in CRF07_BC virus.

Increased folds of EC_50_				
>20	4–20		<4	
V106M	A98G*		(V108I)*	
Y181C*	(V108I)**/T386A**	I135T		
	I135T/I382L*	**(I178M)**		
	K238N**/T48I/(I178M)**	V189I		
		**V314A**		
		**I382L**		
			**T386A**	
			**(I178M)/I382V**	

Novel mutations were bold. Initial mutations were labeled with *. Mutations in parentheses appeared in different mutation profiles.

Drug resistant effects of the 5 reported mutations in CRF07_BC virus were further compared with which were reported in Stanford University HIV drug resistance database (Table 4). Mutations V106M, V108I and Y181C in CRF07_BC were associated with similar NVP resistance as that reported in the database, but A98G in CRF07_BC might be related to higher NVP resistance than that reported in the database. Furthermore, drug resistant effect of K238N is reported to be unknown in the database, but it showed low level of drug resistance in this study.

**Table 4:** Comparison of drug-resistant effects between reported mutations selected in this study and those in HIV drug resistance database of Stanford University.

Drug	Mutations	Level of resistance (increased folds of EC_50_)	
		In this study	In HIV drug resistance database of Stanford University
NVP	A98G	M (17)	L (2–3)
	V106M	H (22–32)	H (70)
	V108I	L (3)	L (UD)
	Y181C	H (450)	H (123)
	K238N	L	Unknown

H: high level of resistance (Fold change (FC)>20); M: medium level of resistance (20>FC>4); L: low level of resistance (FC<4). UD: undetected. They were defined according to Stanford database http://hivdb.stanford.edu/cgi-bin/Marvel.cgi.

## Discussion

Since there is no experimental and clinical data for NVP resistant mutations of HIV CRF07_BC available yet, we designed and performed an *in vitro* NVP selection study. Considering that the results obtained from this study should be used for hypothetical management of RTI treatments for HIV patients infected by this CRF of the virus, we selected a clinical isolate of CRF07_BC virus strain CNGZD, which was isolated from a HIV-1 infected patient, to represent CRF07_BC. NVP was selected for this study, because it has been included in the free ARV treatment in China and currently used in AIDS patients.

The genome of CNGZD was sequenced to understand its genetic background (Table 1, Figure 1, 2, and 3). CRF07_BC was mainly from subtype C with 10 breakpoints and four portions of subtype B’ in gag-pol region, vpr gene, 3′ end of vpu gene and nef gene. The subtype B’ segment in RT was at residues 144–243, containing two of three highly conserved aspartate codons in active sites of the enzyme. The residues 100–200 of RT plays the role as polymerase catalytic site and serves as binding sites of reverse transcriptase inhibitors (RTIs) [26]. The combination of subtype B and C in RT, the key enzyme of HIV, may potentially result in different genotypic and phenotypic drug resistance in CRF_BC virus. It inspired us to explore the drug resistant mutations in RT of this unique CRF.

Primarily isolated virus strain and biological clones of CNGZD were cultured in PBMCs with increasing concentration of NVP. We cannot exclude the possibility of selecting pre-existing minority drug resistant variants from the quasi-species which were not revealed by the Sanger-based population sequencing. However, it has been reported that the pre-existing virus variants will emerge soon after 1 or 2 passages by *in vitro* selection [27]. In our study, NVP-related virus variants were selected after passage 5 and virus strain CNGZD was isolated from an ART treatment naive patient. Thus, the probability of selecting pre-existing minority NVP-resistant variants is quite low. The selection experiment was terminated at 25 passages with 64 µM of NVP because 1) the virus titer of resistant variants in passage 20 to 25, which cultured with 64 µM NVP, was less than 10–20% of the untreated control, so that it was hard to scale up the drug concentration after a few passages of culture with the same NVP concentration; 2) the 64 µM NVP was also reported as the end point of the other *in vitro* selection study [28]. Genotyping and phenotyping of virus variants were performed in different passages of five parallel cultures, respectively. Population sequencing was adopted for genotyping and it has been reported that this Sanger-based sequencing is able to reveal the majority viruses up to 80–90% of the whole population [29]. The mutation profiles and their resistance effects could be correlated because genotypes and phenotypes of virus variants in serial passages of each parallel culture were consecutive and consistent.

NVP resistance mutations are complicate and variable. Thus far, about 32 NVP-resistance associated mutations in 17 positions, including 15 major NVP-resistant mutations at 5 positions (K103NST, V106AM, Y181CIV, Y188LHC, G190ASEQ), have been summarized in HIV drug resistance database of Stanford University [30], [31]. Y181C, K103N, G190A, H221Y, K101E, A98G, V108I, V106A, E138A, Y188L are the top ten most common mutations resistant to NVP in patients who received NVP treatment [32]. Two mutations, V106M and Y181C, have been reported to be major drug resistant mutations [32]. According to Mutation ARV Evidence Listing (MARVEL), V106M appeared in 14% NNRTI treated patients with subtype C HIV infection, whereas it was found in only 0.5% of the treated patients carrying subtype B virus [33]. It seemed that V106M were inclined to develop in subtype C virus. Interestingly, V106M development bias was only found under EFV but not NVP treatment in the previous studies and there was no significant difference of V106M prevalence between subtype B and C in patients receiving NVP treatment [34], [35]. In this study, V106M developed in three out of five parallel cultures. This may be due to those residues 143–200 of RT in CRF07_BC are from subtype B, while the other part of RT, including amino acid 106, are from subtype C. CRF07_BC may inherit V106M development preference from subtype C by NNRTI treatment. As shown in MARVEL, the prevalence of the other major mutation Y181C in NNRTI treated patients with subtype B and C virus are 21% and 12%, respectively. This indicated that both subtype B and C viruses were likely to develop Y181C after receiving NNRTI treatment. In the present study, Y181C was developed in one of five parallel cultures, suggesting that this major drug resistant mutation would also appear in patients infected with CRF07_BC virus after receiving NVP therapy.

The other two mutations, A98G and V108I, have been reported to be polymorphic accessory mutations in patients with subtype B or C virus, while K238N is rarely selected by NNRTI and not common to be found in NNRTI-treated patients with infection of subtype B or C virus (prevalence<0.5%) [33]. Furthermore, seven novel mutations in 6 positions of RT were selected in this study. Polymorphisms at position 4, 48, 135, 178 of RT have been reported in CRF07_BC in China but only I135T was found in treatment naïve patients with CRF07_BC infection [5], [36]. It has been reported that the polymorphic rate in the first 255 codons of RT in CRF07_BC was nearly 3 fold higher than that in subtype B [36]. Consistently, all 7 reported mutations and 3 of 7 novel mutations selected in this study appeared in positions within the first 255 codons of RT. Since the residues 144–243 in RT of CRF07_BC were from subtype B and the residues 100–200 of RT serves as binding sites of RTIs [26], highly frequent genetic variations of CRF07_BC in this region of RT may be associated with rapid and/or novel development of drug resistance in this recombinant virus.

NVP selected variants were defined to 4 combination profiles based on the initial mutations appearing in the selection period. Initial mutations A98G, V108I and Y181C appeared during the first 10 passages in the presence of 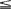
800 nM NVP (4-fold of EC_50_), while double mutations I135T/I382L were detected between passages 10 and 15 (Figure 4). In previous *in vitr*o studies, both Y181C and V106A have been reported as initial mutations resistant to NVP in both subtype B and C viruses [22], [27], [28], [35], but V106A was not an initial mutation selected by NVP treatment in this study. Similarly, V108I has been found in both subtype B and C viruses, whereas A98S was only detected in subtype C virus [35]. The low mutation barrier at these positions suggests that A98G, V108I and Y181C may be found in NVP treated patients infected with CRF07_BC with high possibility. Furthermore, some mutations are likely to appear after the emergence of certain mutations. For example, V108I has been reported to correlate with the appearance of Y181C in the patients or in cell culture as an accessory mutation [32], [35] and it also recurred in the present study. Our results also showed that V106M was likely to accumulate with I178M in CRF07_BC. The complex NNRTI mutations developed in CRF07_BC *in vitro* selection may be highly possible to occur in clinics.

In summary, this study has showcased the evolution of virus resistance to NVP in clinical isolated CRF_07BC virus by *in vitro* PBMC culture system. Our study identified 7 novel mutations and 7 reported mutations selected by NVP treatment that are associated with different levels of drug resistance to NVP. The results have also suggested that major NNRTI mutations Y181C and V106M may be found in patients harboring CRF07_BC virus with high possibility when NVP was included in ART treatment and these two mutations may cause serious resistance to not only NVP but also other NNRTI. Furthermore, novel mutations have been selected by NVP treatment in diverse and variable combination with the other major mutations in our study. This phenomenon may happen when more and more patients infected with different HIV CRFs come into therapy. Although the information obtained in this *in vitro* selection study will not fully cover the resistant mutations which will actually occur in patients, it has still provided useful information for rational management of ART in patients infected with HIV CRF_07BC subtype.
